# Urinary Catheters Coated with a Novel Biofilm Preventative Agent Inhibit Biofilm Development by Diverse Bacterial Uropathogens

**DOI:** 10.3390/antibiotics11111514

**Published:** 2022-10-30

**Authors:** Stephany Navarro, Ethan Sherman, Jane A. Colmer-Hamood, Thomas Nelius, Matthew Myntti, Abdul N. Hamood

**Affiliations:** 1 Department of Immunology and Molecular Microbiology, Texas Tech University Health Sciences Center, Lubbock, TX 79430, USA; 2 Next Science, Jacksonville, FL 32256, USA; 3 Department of Medical Education, Texas Tech University Health Sciences Center, Lubbock, TX 79430, USA; 4 Department of Urology, Texas Tech University Health Sciences Center, Lubbock, TX 79430, USA; 5 Department of Surgery, Texas Tech University Health Sciences Center, Lubbock, TX 79430, USA

**Keywords:** antibiofilm agent, biofilm, catheter coating, *Escherichia coli*, *Pseudomonas aeruginosa*, silicone catheter, urinary catheter, urinary tract infection, uropathogen

## Abstract

Despite the implementation of stringent guidelines for the prevention of catheter-associated (CA) urinary tract infection (UTI), CAUTI remains one of the most common health care-related infections. We previously showed that an antimicrobial/antibiofilm agent inhibited biofilm development by Gram-positive and Gram-negative bacterial pathogens isolated from human infections. In this study, we examined the ability of a novel biofilm preventative agent (BPA) coating on silicone urinary catheters to inhibit biofilm formation on the catheters by six different bacterial pathogens isolated from UTIs: three *Escherichia coli* strains, representative of the most common bacterium isolated from UTI; one *Enterobacter cloacae,* a multidrug-resistant isolate; one *Pseudomonas aeruginosa,* common among patients with long-term catheterization; and one isolate of methicillin-resistant *Staphylococcus aureus*, as both a Gram-positive and a resistant organism. First, we tested the ability of these strains to form biofilms on urinary catheters made of red rubber, polyvinyl chloride (PVC), and silicone using the microtiter plate biofilm assay. When grown in artificial urine medium, which closely mimics human urine, all tested isolates formed considerable biofilms on all three catheter materials. As the biofilm biomass formed on silicone catheters was 0.5 to 1.6 logs less than that formed on rubber or PVC, respectively, we then coated the silicone catheters with BPA (benzalkonium chloride, polyacrylic acid, and glutaraldehyde), and tested the ability of the coated catheters to further inhibit biofilm development by these uropathogens. Compared with the uncoated silicone catheters, BPA-coated catheters completely prevented biofilm development by all the uropathogens, except *P. aeruginosa*, which showed no reduction in biofilm biomass. To explore the reason for *P. aeruginosa* resistance to the BPA coating, we utilized two specific lipopolysaccharide (LPS) mutants. In contrast to their parent strain, the two mutants failed to form biofilms on the BPA-coated catheters, which suggests that the composition of *P. aeruginosa* LPS plays a role in the resistance of wild-type *P. aeruginosa* to the BPA coating. Together, our results suggest that, except for *P. aeruginosa*, BPA-coated silicone catheters may prevent biofilm formation by both Gram-negative and Gram-positive uropathogens.

## 1. Introduction

Complicated urinary tract infections (UTIs) involve conditions that compromise the urinary tract and/or its host defenses, including the presence of foreign bodies within the urinary tract, such as indwelling catheters and bladder or renal calculi, renal failure, and immunosuppression [1,2]. About 80% of complicated UTIs are related to indwelling catheters [3]. Indwelling urinary catheters, which are a closed system consisting of the catheter tubing, a small retention balloon, and a drainage collection bag, are inserted through the urethra or suprapubically to allow for bladder drainage [4]. Unfortunately, catheter usage, especially prolonged use, often leads to catheter-associated UTIs (CAUTIs) that arise when bacteria enter the urinary tract via insertion of the catheter, or during post-placement manipulation of the catheter or its drainage system [5,6,7]. Catheters also provide a surface to which bacteria can adhere and develop biofilms [7]. CAUTIs are among the most frequent types of hospital-acquired infections, currently ranking second [8,9]. It is estimated that, in the U.S., more than 30 million bladder catheters are annually inserted, with several hundred thousand CAUTIs associated with those insertions [10]. CAUTIs may lead to potentially serious consequences, including frequent febrile episodes, acute and chronic pyelonephritis, bacteremia with urosepsis and septic shock, catheter obstruction and renal and/or bladder stone formation, and even bladder cancer with prolonged use [11,12,13]. Placement of a urinary catheter led to a 39-fold increase in bacteremia in one study [11]. Of bloodstream infections noted 48 h or more after admission, 21% were from a urinary source and 71% of those were catheter-associated [12]. In long-term care facilities, CAUTIs have been estimated to be the source of 45–50% of bacteremia [14,15]. The most common pathogens associated with CAUTI include uropathogenic *Escherichia coli*, *Klebsiella* spp., *Pseudomonas aeruginosa*, *Enterococcus faecalis*, and other *Enterobacterales* [9,11,16].

Pathogens associated with CAUTIs are increasingly resistant to antimicrobial agents. In both acute and long-term care facilities, the urine of patients with indwelling catheters is the main source of resistant Gram-negative bacteria, including extended spectrum beta-lactamase (ESBL), producing *Enterobacterales* and carbapenem-resistant *Enterobacterales* (CRE) [17,18]. Uropathogens frequently form biofilms first on the surfaces (interior and/or exterior) of the indwelling catheter, and then on the uroepithelium and drainage bag [11,13]. These catheter-associated biofilms, which consist of the attached bacteria, their extracellular products, and host components, provide the uropathogens with many survival advantages, including resistance to the sheer force of the urine flow, resistance to phagocytosis, and resistance to antimicrobial agents [11,19,20,21]. Therefore, an ideal urinary catheter is one coated with a strong antimicrobial agent that prevents the uropathogens from colonizing the catheter and developing antibiotic-resistant biofilms. Multiple antimicrobial urinary tract catheter coating agents have been described [22] with variable effectiveness, including silver alloy [23], chlorhexidine [24,25], triclosan [24,26], antibiotics [23,27], antimicrobial peptides [28], and bacteriophages [29]. A meta-analysis of indwelling urethral catheters concluded that silver alloy-coated catheters were not associated with a statistically significant reduction in symptomatic CAUTI, and that while use of a nitrofurazone catheter reduced the risk of symptomatic CAUTI, the patients experienced more discomfort with these catheters [30]. It was further noted that several previously available antiseptic-coated and antimicrobial-coated catheters are no longer available [30]. Coatings that prevent bacterial adhesion to the catheter yet have no antimicrobial activity have been investigated, also with varying results: hydrogel- and polytetrafluoroethylene-coated urinary catheters are commercially available, while other compounds such as polyzwitterion polymers, polyethylene glycol, and enzymes (the quorum-sensing inhibitor acylase, and exopolysaccharide-specific glycoside hydrolase) have shown promising results in research [22,31]. The development of antimicrobial coatings for urinary catheters has been ongoing, with reports of successful killing of *P. aeruginosa* for up to 30 days by a catheter designed to release ciprofloxacin and azithromycin [32]; killing of *Proteus mirabilis*, *Staphylococcus aureus*, and *E. coli* for 7–12 weeks by slow release of rifampicin, sparfloxacin, and triclosan impregnated in a catheter [33]; and prevention of biofilm formation by *S. aureus* and *E. coli* on catheters coated with different acrylate compounds and the killing of these organisms by the release of antibiotics (rifampicin, sparfloxacin, and triclosan) impregnated within the coating [34].

We previously demonstrated the effectiveness of a biofilm dispersal formulation developed by the Next Science company (NS, when in conjunction with the different antimicrobial/antibiofilm agents tested) (Jacksonville, FL, USA), inhibiting the growth and biofilm development by different Gram-positive and Gram-negative pathogens isolated from wound and middle ear infections [35,36]. Additionally, we assessed the NS-formulated wound solution Bactisure in inhibiting biofilm development by an *E. coli* laboratory strain and three *E. coli* clinical isolates on three types of urinary catheters [37]. When added to the growth medium containing catheter pieces, NS Bactisure inhibited biofilm development by all four *E. coli* strains on each of the three catheter types [37]. However, for practical application, it is essential to coat urinary catheters with a specific NS formulation and examine the effectiveness of the coated catheter in preventing biofilm formation. In this study, we report an examination of biofilm development by eight bacterial strains isolated from UTIs on silicone urinary catheters coated internally and externally with a novel NS biofilm preventative agent (BPA). To provide additional relevance, biofilm development by the uropathogens was tested on BPA-coated catheters incubated in a medium that closely mimics human urine.

## 2. Results

### 2.1. E. coli Predominated among the UTI Isolates

**Figure 1:**
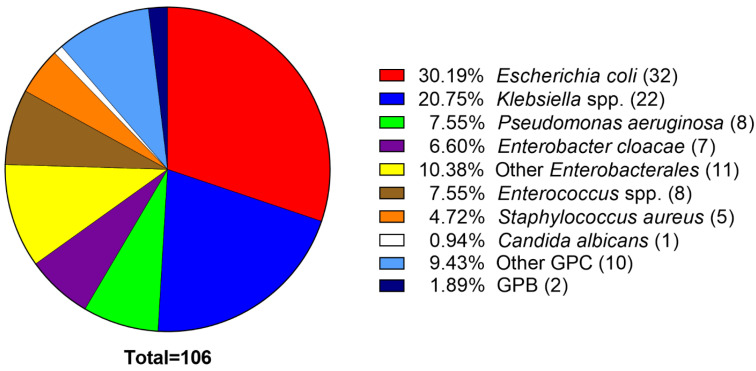
Distribution of the 106 uropathogenic isolates. Numbers in parentheses indicate numbers of isolates. *Klebsiella* spp. includes *K. pneumoniae* (18), *K. oxytoca* (2), and *K. aerogenes* (2); other *Enterobacterales* includes *Serratia marcescens* (3), *Citrobacter freundii* (3), *C. koseri* (2), *Proteus mirabilis* (2), and *Hafnia alvei* (1). *Enterococcus* spp. includes *E. faecalis* (6) and *E. faecium* (2). Other GPC isolates include *Streptococcus agalactiae* (5), *S. anginosus* (1), *Staphylococcus epidermidis* (2), *Aerococcus urinae* (2); GPB isolates are *Lactobacillus* sp. (1) and *Actinomyces neuii* (1). GPC, Gram-positive cocci; GPB, Gram-positive bacilli.

Through a protocol approved by the Institutional Review Board at Texas Tech University Health Sciences Center (TTUHSC), we obtained 106 clinical isolates from urine samples of patients, many with long-term catheterization, presenting with UTI at the TTUHSC Urology Clinic. The most frequently isolated bacterial species were *E. coli* (32) followed by *Klebsiella* spp. (22), *P. aeruginosa* (8), and *Enterobacter cloacae* (7) (Figure 1). We then compared the distribution of our isolates to the most common agents of CAUTI, as reported by the National Healthcare Safety Network (NHSN) database for 2015–2017, containing 85,545 isolates from hospitalized patients [9], to determine how closely our outpatient isolates matched those of the hospitalized patients. By ranking, our first three, *E. coli*, *Klebsiella* spp., and *P. aeruginosa*, were the same as the national database [9]. The percentages of *E. faecalis* and *Proteus* spp. isolates among our collection were less (5.7% vs. 9.3% and 1.9% vs. 5.6%) while those of *Enterobacter* spp. and *S. aureus* were greater (6.6% vs. 4.9% and 4.7% vs 2.1%, respectively) (Figure 1) [9]. Additionally, our isolates included 4.7% *Streptococcus agalactiae* and 0.9% *Candida albicans*, while the inpatient database did not include either of those microorganisms among the top 15 species isolated (Figure 1) [9].

### 2.2. Several UIs Exhibited Multidrug Resistance

As antibiotic resistance has been increasing in general and among isolates from CAUTIs [17,18], we selected six isolates for which we obtained antibiotic susceptibility results for further examination: three *E. coli* isolates (UI-033-EsC, UI-038-EsC, and UI-061-EsC), representative of the most common agent of CAUTI; one *E. cloacae* (UI-095-EnC); one *P. aeruginosa* (UI-092-PA), a common cause of CAUTI in long-term catheterized patients; and one methicillin resistant *S. aureus* (UI-009-MRSA), to represent Gram-positive uropathogens. The three *E. coli* isolates and the *E. cloacae* isolate exhibited patterns of resistance that were quite similar (Table 1). Isolate UI-038-EsC was the most resistant among the *E. coli*, fully susceptible to only six of the twenty-three tested drugs (26%); UI-095-EnC was most resistant overall—susceptible to five of the twenty-one drugs tested (24%) (Table 1). Defining multidrug resistance (MDR) as an isolate that is resistant to three or more classes of antibiotics or that carries a key resistance marker (such as methicillin resistance for staphylococci or ESBL production for the *Enterobacterales*) [38,39], isolates UI-033-EsC, UI-038-EsC, UI-061-EsC, and UI-095-EnC were MDR and ESBL *Enterobacterales*, but none produced carbapenemase (Table S1 in Supplementary Materials). Of the ten drug classes tested, UI-038-EsC, UI-061-EsC, and UI-095-EnC were resistant to seven: cephalosporins, fluoroquinolones, monobactams, penicillins, sulfonamides, tetracyclines, and aminoglycosides (the *E. coli* isolates) or nitrofurans (the *E. cloacae* isolate) (Table S1 in Supplementary Materials). The only class of antibiotics for which no resistance was noted among any of the *Enterobacterales* isolates was the glycylcyclines (Table 1 and Table S1 in Supplementary Materials).

UI-092-PA was resistant to imipenem, but was susceptible to meropenem and the other beta-lactams tested (Table 1). Carbapenem resistance in such isolates is usually due to inactivation of the OprD porin, rather than production of carbapenemase [40,41]. The strain was not considered MDR as it showed intermediate susceptibility to the fluoroquinolones, and susceptibility to all of the other classes (Table S1 in Supplementary Materials). UI-009-MRSA was MDR due to the presence of methicillin resistance, even though it was resistant only to one other class of antibiotics—the macrolides, via inducible clindamycin resistance (Table 2 and Table S2 in Supplementary Materials) [38,39,42]. Of the three other *S. aureus* isolates for which susceptibilities were available, none were resistant to methicillin, but UI-073-SA and UI-078-SA were resistant to three classes of antibiotics and considered MDR, while UI-096-SA was susceptible to at least one drug in each class of antibiotics (Table S2 in Supplementary Materials). Analysis of eight *Enterococcus* spp. Isolates showed only two were MDR: *E. faecalis* isolate UI-065-Efc was resistant to three of eight classes of antibiotics tested and *E. faecium* isolate UI-048-Efm showed resistance to four classes (Table S3 in Supplementary Materials).

### 2.3. Artificial Urine Medium Supports Growth and Biofilm Development by the Uropathogens

**Figure 2:**
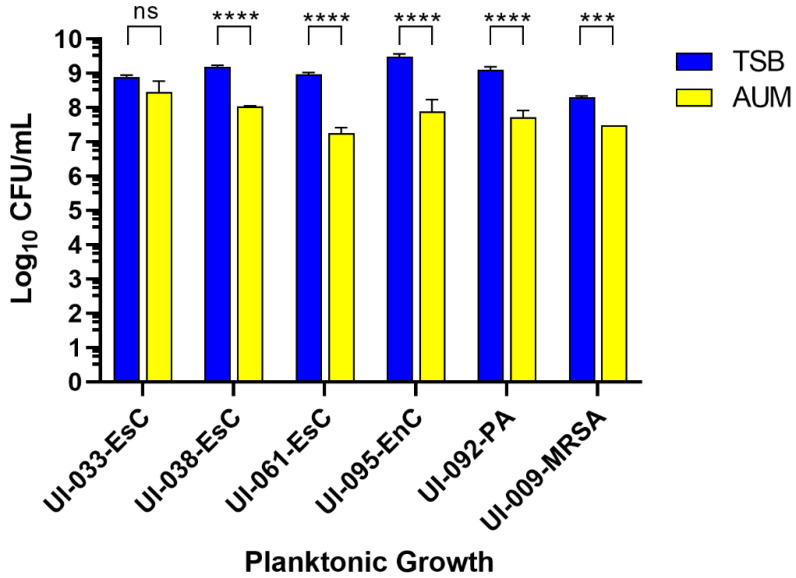
Artificial urine medium supports the growth of clinical uropathogens. Clinical isolates were grown in the microtiter plate assay to assess planktonic growth in tryptic soy broth (TSB) or artificial urine medium (AUM). Colony-forming units (CFU) were determined after 24 h at 37°C. Data were transformed to log_10_ before graphing. Bars represent the average of three independent experiments ± SEM; ns, no significance; *** *p* < 0.001, **** *p* < 0.0001.

To more closely mimic bacterial growth within the urinary bladder, we used artificial urine medium (AUM), which is similar to human urine [43]. This medium was previously shown to not only support the growth of Gram-positive and Gram-negative uropathogens, but to also promote crystal production by *Staphylococcus epidermidis* and *P. mirabilis* [43]. After 24 h of growth at 37 °C ± 0.5 °C, and compared to their growth in the rich medium TSB, the growth of five of the six uropathogens was significantly reduced in AUM (Figure 2). Only UI-033-EsC grew at a comparable level in both media. The observed reduction in growth in AUM was expected since the AUM is a chemically-defined medium with restricted nutrients. While growth was not as robust in AUM, the medium did support sufficient growth of the uropathogens for subsequent experiments (10^7^ to 10^9^ CFU/mL) (Figure 2). 

**Figure 3:**
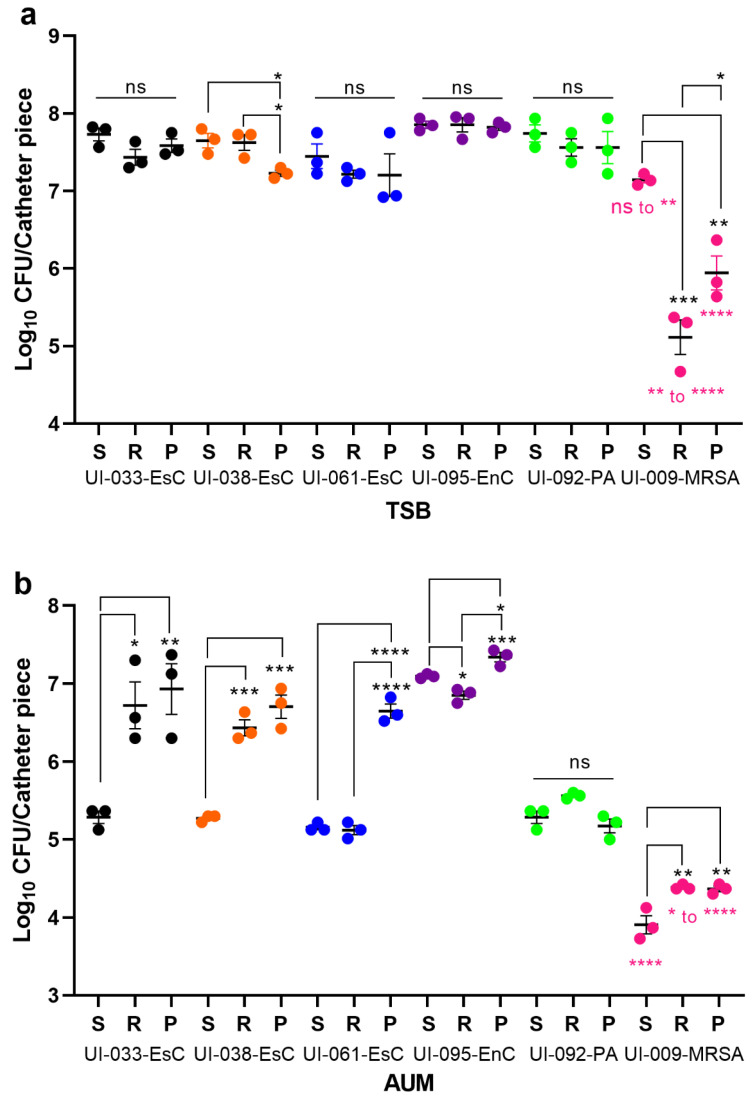
Artificial urine medium supports the growth of clinical uropathogens and their formation of biofilm on urinary catheters. Biofilms were allowed to develop for 24 h on 1.5-cm lengths of urinary catheters made of silicone (S), rubber (R), or PVC (P) incubated in TSB (**a**) or AUM (**b**). The amounts of biofilm formed by each isolate on each type of catheter material were compared. For five of the six isolates, biofilm biomass on silicone is significantly less than that formed on PVC; for four of the six isolates, biofilm biomass on silicone is less than that formed on rubber. Each dot represents an independent experiment; ns, no significance; * *p* < 0.05; ** *p* < 0.01; *** *p* < 0.001, **** *p* < 0.0001. Black asterisks indicate significant differences for each isolate and the different catheter materials; magenta asterisks indicate significant differences in biofilm biomass of UI-009-MRSA on each type of catheter material compared to the other bacteria.

The ability of the uropathogens to form biofilm on urinary catheters when grown in AUM was assessed using the microtiter plate biofilm assay. Urinary catheters are made of different materials, with the most common being silicone, rubber, and polyvinyl chloride (PVC). Urinary catheters made of these three materials were aseptically cut into 1.5-cm lengths and individual pieces were inoculated with the uropathogens in TSB or AUM. After 24 h at 37 °C ± 0.5 °C, all six uropathogens formed biofilms on each type of catheter when grown in TSB (Figure 3a). Except for UI-009-MRSA, which produced biofilms with significantly less biomass than the other strains on all three types of catheters, the biomass of the biofilms formed by the Gram-negative bacilli was similar: an average of 7.7 logs/catheter piece on the silicone catheter, 7.5 logs on the rubber catheter, and 7.5 logs on the PVC catheter (Figure 3a). In AUM, the amount of biofilm formed by all tested uropathogens on the three surfaces was reduced from that produced in TSB (Figure 3b); similar to the reduction in planktonic growth in AUM vs. TSB (Figure 2). The average biomass of all the biofilms formed in AUM on the silicone catheters was 5.3 logs/catheter piece, with 5.8 logs/catheter piece on the rubber catheters and 6.9 logs/catheter piece on the PVC catheters (Figure 3b). Again, the biomass of UI-009-MRSA was consistently and significantly less than that produced by the other isolates, regardless of surface (Figure 3b). The largest reduction in overall biomass, from 7.7 logs in TSB to 5.3 logs in AUM, was seen with the silicone catheters (Figure 3b), which substantiates reports that silicone catheters undergo less bacterial colonization in vivo [44,45]. Comparison of biofilm biomass formed by each isolate grown in AUM on the three types of catheters showed that the biomass of biofilms formed by UI-033-EsC, UI-038-EsC, and UI-009-MRSA on silicone was less than that formed on rubber or PVC (Figure 3b). The biomass of biofilms formed by UI-061-EsC and UI-095-EnC were less on silicone than on PVC, and that of UI-092-PA was less than that on rubber (Figure 3b). Direct comparison of biofilm biomass of each UI on each type of catheter material when grown in TSB versus AUM showed that the reduction in biomass was most consistent and most significant over all the Uis when silicone catheter material was tested (Figure S1 in Supplementary Material). Since our goal was to reduce bacterial colonization of the urinary catheter, we decided to continue with the silicone catheters.

### 2.4. BPA-Coated Catheters Inhibit Biofilm Development by the Uropathogens

**Figure 4:**
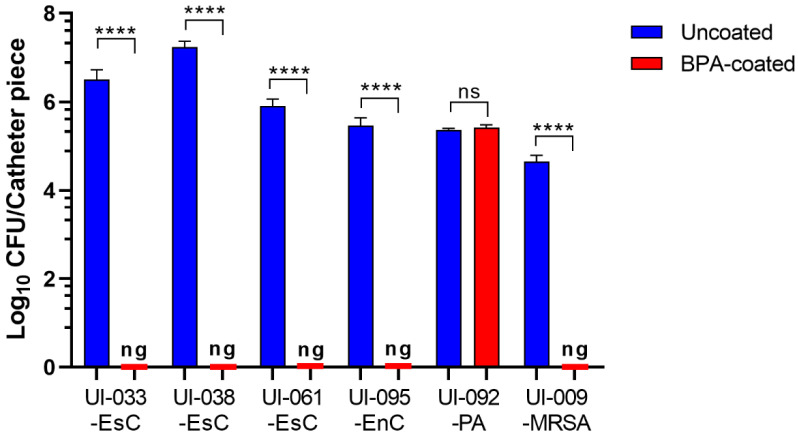
BPA-coating on silicone catheters inhibits biofilm formation by most of the uropathogens. The BPA coating was not effective in preventing biofilm formation by UI-092-PA. Values represent the average of three independent experiments ± SEM; ns, no significance; **** *p* < 0.0001.

It is estimated that 70%-80% of UTIs are associated with catheter use [3]. Thus, coating catheters with antimicrobial agents is an essential approach to prevent UTI. Multiple antimicrobial agents have been used to coat urinary catheters with varying degrees of reported success [46,47]. We previously reported successful inhibition of biofilm formation by several *E. coli* clinical isolates when urinary catheters were treated with NS Bactisure (0.13% benzalkonium chloride [BZK], sodium acetate, acetic acid, ethanol, and water; per list on package) [37]. Thus, for the next step, Next Science coated 1.5-cm lengths of silicone urinary catheters with BPA (0.175% BZK, 1.99% polyacrylic acid, 2.62% glutaraldehyde, and 95.215% sterile water for irrigation) and returned the coated pieces for determination of the ability of the BPA-coated catheters to prevent biofilm formation by uropathogens. Except for *P. aeruginosa*, BPA-coated catheters inhibited biofilm development by all tested uropathogens (Figure 4).

**Figure 5:**
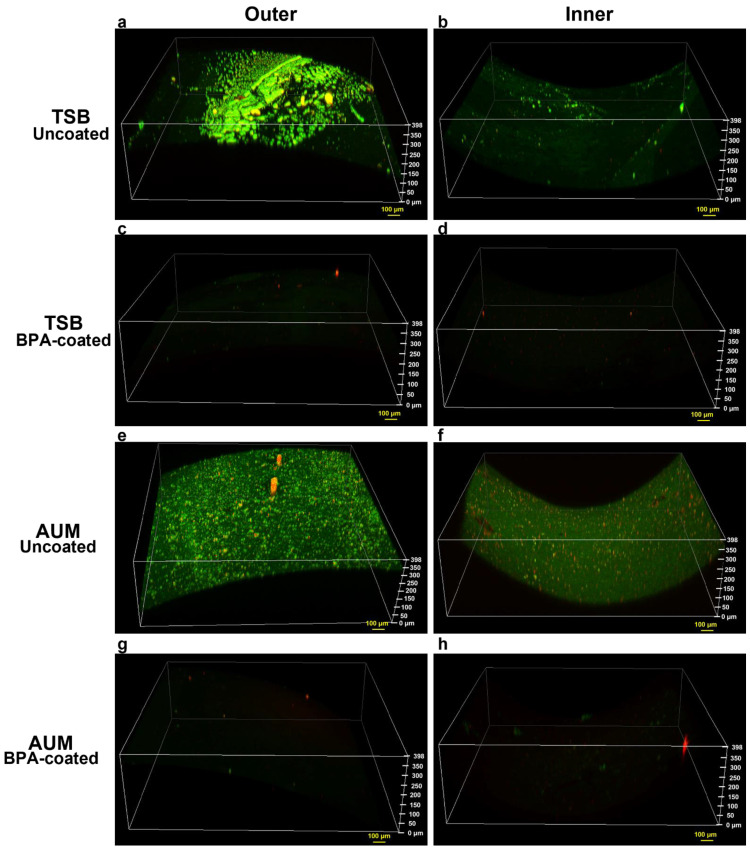
BPA-coating inhibits biofilm formation by UI-033-ESC on the inner and outer surfaces of the coated catheter. Biofilm development was visualized using CSLM following staining with the LIVE/DEAD *Bac*Light™ Bacterial Viability kit; live bacteria are stained green and dead bacteria are stained red. Outer (**a**) and inner (**b**) surfaces of uncoated catheters and outer (**c**) and inner (**d**) surfaces of BPA-coated catheters incubated in TSB. Outer (**e**) and inner (**f**) surfaces of uncoated catheters and outer (**g**) and inner (**h**) surfaces of BPA-coated catheters incubated in AUM. Yellow bars = 100 μm; images are representative of three independent experiments.

We confirmed the inhibition of biofilm development by UI-033-EsC by CLSM. Biofilms formed on the inner and outer surface of the catheter pieces were visualized by staining with LIVE/DEAD *Bac*Light™ staining. UI-033-ESC formed biofilms on both sides of the catheter pieces, whether grown in TSB (Figure 5a,b) or AUM (Figure 5e,f). As expected from the more robust biofilm development by UI-033-EsC in TSB compared to AUM (Figure 3), the biofilms formed on both surfaces of the catheter pieces incubated in TSB appear to have more structure than those formed in AUM (Figure 5a,b,e,f). In contrast, we detected no attached bacteria on either surface of the BPA-coated catheter pieces following growth in TSB or AUM (Figure 5c,d,g,h). To confirm these results, we also visualized biofilms formed by UI-038-EsC; similar results were observed—structured biofilm development on outer and inner surfaces of the uncoated catheters when incubated in TSB and less robust biofilm formation in AUM while the BPA-coated catheters showed no biofilm present in TSB or AUM (Figure S2 in Supplementary Material). These results strongly suggest that the BPA coating is effective in preventing biofilm development by bacterial uropathogens on both surfaces of urinary catheters.

### 2.5. Lipopolysaccharide Contributes to the Tolerance of P. aeruginosa to the BPA-Coated Catheters

**Figure 6:**
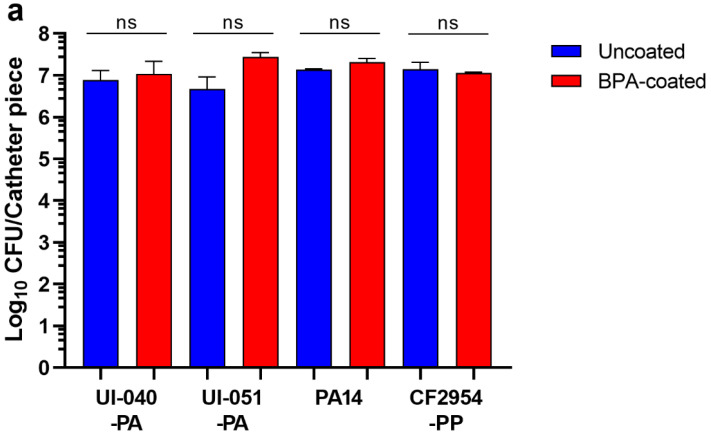

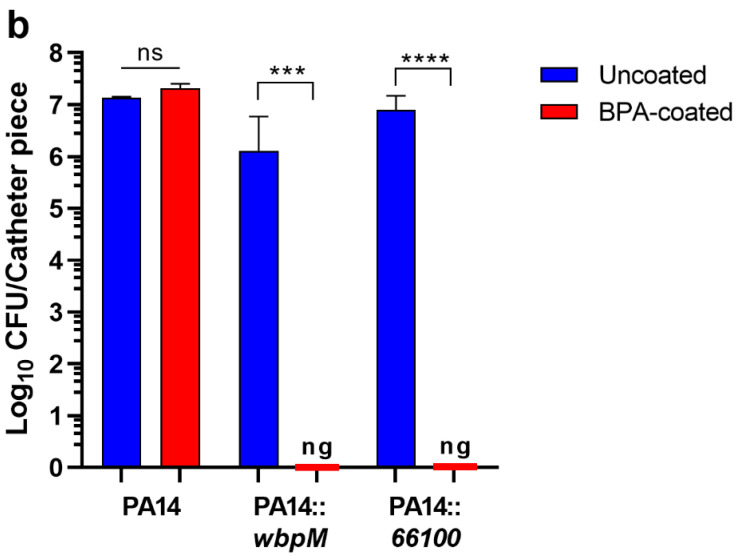
*Pseudomonas* species are tolerant to BPA-coated catheters. (**a**) Uncoated and BPA-coated catheters were placed in AUM inoculated with two additional *P. aeruginosa* UTI isolates (UI-040-PA, UI-051-PA), the virulent *P. aeruginosa* laboratory strain (PA14), and a respiratory isolate of *P. putida* (CF2954-PP). (**b**) Isogenic LPS mutants of PA14 are sensitive to the BPA-coated catheters. Uncoated and BPA-coated catheters were placed in AUM inoculated with PA14 (parent strain) and its isogenic mutants PA14::*wbpM* and PA14::*66100*. Bars represent the average of three independent experiments ± SEM; ns, no significance; *** *p* < 0.001; **** *p* < 0.0001.

The BPA-coated catheters not only failed to inhibit UI-092-PA biofilm development, but the biofilm biomass was also not reduced (Figure 4). To investigate the possibility that this phenomenon is limited to this *P. aeruginosa* strain only, we examined the effectiveness of the BPA-coated catheter pieces in inhibiting biofilm development by two other *P. aeruginosa* uropathogens. Biofilm development by UI-040-PA and UI-051-PA on uncoated and BPA-coated catheter pieces was the same (Figure 6a). We then assessed the effect of the BPA-coated catheters on biofilm development by the virulent *P. aeruginosa* strain UCBPP-PA14 (PA14), which has been utilized extensively in various in vitro and in vivo studies [48,49,50,51,52]. Again, BPA-coated catheter pieces did not affect biofilm development by this strain (Figure 6a). This phenomenon was not limited to only *P. aeruginosa*; BPA-coated catheters also failed to prevent biofilm development by a *P. putida* clinical isolate (CF2945-PP) (Figure 6a). One component possibly contributing to *Pseudomonas* resistance to killing by the BPA-coated catheter (while the other Gram-negative bacilli were susceptible) could be its lipopolysaccharide (LPS). It has been previously shown that in contrast to *E. coli*, *P. aeruginosa* is more tolerant to the antibiotic colistin due to the release of large amounts of LPS [53]. To explore the possibility that LPS is a contributing factor, we examined two PA14 mutants deficient in the LPS O-antigen (PA14::*wbpM* and PA14::*66100*) [54]. Unlike the parent strain PA14, both mutants were sensitive to the BPA coating (Figure 6b), which suggests that *P. aeruginosa* tolerance to the BPA coating is due, at least in part, to its LPS.

## 3. Discussion

CAUTIs are caused by many different bacterial and fungal pathogens, whose prevalence varies according to their location of isolation. In this study, we categorized a pool of 106 Gram-positive and Gram-negative uropathogens from outpatients at the TTUHSC Urology Clinic. Similar to other reports, *E. coli* was the most common isolate at 30% (Figure 1) [9,55,56]. While the percentages and types of organisms isolated varied from the data reported by the NHSN [9], our data was quite similar to those reported by Khan et al., who analyzed a combination of inpatient and outpatient specimens: 30% *E. coli*, 21% *Klebsiella* spp., and 8% *P. aeruginosa* (Figure 1) vs. 36%, 21%, and 9%, respectively [55]. 

We were able to obtain the antibiotic resistance profiles for the five Gram-negative bacilli tested, four *S. aureus* isolates including UI-009-MRSA, and eight *Enterococcus* spp. (Table 1, Table 2 and Tables S1–S3 in Supplementary Materials). Nine of the seventeen strains met the definition of MDR pathogens. Of the six isolates selected for testing, all of the *E. coli* isolates, the *E. cloacae* isolate, and the MRSA isolate were MDR. Surprisingly, the *P. aeruginosa* isolate, which was resistant to the BPA coating, was not MDR. Despite their resistance to many different classes of antibiotics, the remaining uropathogens tested were sensitive to the BPA-coated silicone catheters (Figure 4), suggesting that BPA is a promising broad-spectrum antimicrobial coating agent. However, to support such an assumption, we need to test the sensitivity of the remaining 100 uropathogens.

Whether bacteria enter during insertion of the catheter or afterwards via the sheath of exudate associated with the catheter (extraluminally), or travel intraluminally along the tubing between the bladder and the collection bag [5,6,7], attachment of the bacteria to either surface (inner or outer) with subsequent biofilm development is a crucial component for initiation and establishment of CAUTIs [11,13,19,57]. Following attachment to the catheter, the uropathogens can produce thick condensed biofilms containing microbial polysaccharides, extracellular substances, and host products [58,59]. These biofilms, which can occlude the catheter, protect the bacteria from natural host defenses and antibiotic activities [19,59,60]. As a result, systemic antibiotics eradicate planktonic bacteria within the bladder but fail to eliminate the bacteria within the biofilm [59,60,61]. Due to the development of bacterial biofilms on the outer and inner surfaces of the catheter, the best approach to prevent CAUTIs is to coat both surfaces of the catheter with antibiofilm/antimicrobial agents [22]. The recovery of no bacteria from the BPA-coated catheters incubated with *E. coli*, *E. cloacae*, and *S. aureus* suggests that the BPA-coated catheters prevented biofilm formation on both surfaces (Figure 4). This was substantiated by the lack of visible biofilm in the CLSM examination of catheters exposed to two different *E. coli* strains (Figure 5 and Figure S1 in Supplementary Materials).

While approaches to preventing biofilm formation, targeting each stage of development from attachment to maturation and dispersion, have been described [62,63,64,65], the best approach for prevention of CAUTI seems to be preventing attachment in the first place, which is the most likely role of the BPA coating (0.175% BZK) used in this study. We previously showed successful inhibition of biofilm formation by different Gram-positive and Gram-negative pathogens in pre-market testing of NS BlastX wound gel (0.13% BZK, polyethylene glycol 400, polyethylene glycol 3500, sodium citrate, citric acid, and water; per listing on package) and NS Bactisure wound lavage (0.13% BZK) [35,36]. Although their formulations vary, the common active component among these three compounds is BZK, which is a quaternary ammonium compound (QAC) (in this study, PubChem CID 16372, https://pubchem.ncbi.nlm.nih.gov/compound/13762; accessed on 24 October 2022) [66]. QACs are cationic detergents that have been shown to be bactericidal when applied as coatings; essentially killing on contact [64,67,68]. Three mechanisms have been proposed for this contact killing. (1) Penetration of the cell membrane by long cationic polymers [68] or by shorter polymers at high density [69] causes the cell to break open. (2) Electrostatic interaction between the cationic QAC coating and ions on the net negative charge on the bacterial cytoplasmic membrane causes lysis and death [69,70]. (3) Selective adhesion of negatively charged phospholipids in the bacterial cell membrane to the cationic surface causes the leakage of cell contents (the phospholipid sponge effect) [70]. The exact mechanism by which BPA-coated catheters prevent biofilm formation by the different pathogens is not known, but the complete lack of organisms recovered from the biofilm inhibition experiments (Figure 4) suggests that bactericidal contact killing is taking place.

It is unlikely that the additional compounds used to produce the BPA coating made any contribution to the bactericidal contact killing. Polyacrylic acid used in the preparation of the BPA-coated catheters does not have antimicrobial effects (PubChem CID 6581, https://pubchem.ncbi.nlm.nih.gov/compound/6581) (accessed on 24 October 2022) [66], although polyacrylates have been shown to provide broad spectrum resistance to biofilm formation by several different uropathogens [34,71,72]. While the mechanism by which this occurs is not known, it is not due to killing, but is possibly due to the hydrophobicity of polyacrylate and its interaction with the bacterial lipophilic cell wall [71,72]. Polyacrylates can be used to form stable non-leaching coatings [69,73] or they can be used to produce coatings that release antimicrobials impregnated within the coating [34,74]. In contrast, glutaraldehyde, which is also used as chemical intermediate and a cross-linking agent, does have disinfectant properties and has been long used for cold sterilization of heat-sensitive equipment like endoscopes, as a biocide, and as an antimicrobial agent in a number of environments (PubChem CID 3485; https://pubchem.ncbi.nlm.nih.gov/compound/3485) (accessed on 24 October 2022) [66]. As there have been reports of toxicity associated with the use of glutaraldehyde as a disinfectant for endoscopes and other equipment [75,76], glutaraldehyde is unlikely to be an active ingredient.

It is probable that one or more of the three mechanisms described for contact killing (penetration of the cell membrane, electrostatic interaction between the cationic BZK and the negatively charged cell membrane, and selective adhesion of negatively-charged phospholipids to the cationic BZK, [68,69,70]) are involved in the observed prevention of biofilm formation by the MDR strains of *E. coli*, *E. cloacae*, and *S. aureus*. Results from our previous studies suggest that solutions containing BZK are more effective against Gram-positive pathogens than Gram-negative pathogens. Through titration experiments, we previously demonstrated that the minimum inhibitory concentration of such a NS solution (for prevention of biofilm on tympanostomy tubes) for the Gram-negative bacillus *P. aeruginosa* was 16-fold higher than that for the Gram-positive coccus *S. aureus* [35]. Similarly, we recently found that the minimum bactericidal concentrations (MBCs), as well as the minimum biofilm inhibition concentrations (MBfIC) of BZK in NS Bactisure, were about 32-fold less for UI-009-MRSA than those for UI-092-PA, and 8- to 16-fold less than those for UIs of *E. coli* and *E. cloacae*; additionally, the MBfIC for UI-092-PA was two to four times higher than that of *E. coli* or *E. cloacae* (Table S4 and Methods S1 and S2 in Supplementary Materials). Since their cell walls are composed of peptidoglycan and teichoic acid, which are slightly negatively charged, Gram-positive bacteria including *S. aureus* are more readily killed by BZK at lower concentrations than are Gram-negative ones [77]. 

However, the *P. aeruginosa* isolate UI-092-PA, which was the most susceptible to antibiotics, was completely resistant to the BPA coating (Figure 4; Table 2; and Table S1 in Supplementary Materials). The need for a higher concentration of BZK to kill Gram-negative bacteria is due to the presence of lipopolysaccharide (LPS) [78,79,80]. First, the tightly packed LPS molecules considerably slow diffusion of hydrophobic compounds; and second, the presence of divalent cations (calcium and magnesium) within the polyanionic lipid A and the inner core oligosaccharides allow bridging of the negative charges of the LPS molecules, linking them into a tight network [80,81]. Furthermore, the length of the LPS plays a role in susceptibility or resistance to ammonium-based ionic liquids, including QACs; wildtype *E. coli* and an outer core mutant were the least susceptible while inner core mutants showed elevated sensitivity [79]. The LPS of *E. coli* (EsC-LPS) and *P. aeruginosa* (PA-LPS) are structurally different and induce different reactions with the host defensive proteins: EsC-LPS complexed with sheep lung surfactant was more toxic than PA-LPS [82]; PA-LPS bound to human α-1-acid glycoprotein with the greatest affinity compared to EsC-LPS or LPS from *Salmonella enterica* or *Serratia marcescens* [83]; and rat cationic defensins effectively killed all the Gram-negative isolates tested, including EsC-LPS mutants, but failed to kill Gram-positive isolates [84]. Therefore, the LPS of *Pseudomonas* could be responsible for its resistance to BPA (Figure 6a). The observed resistance to BPA among our *P. aeruginosa* isolates was not a phenomenon of global resistance to BZK, as other BZK-containing NS antimicrobial agents effectively inhibited the growth and biofilm development of different *P. aeruginosa* clinical isolates and laboratory strains when applied as a solution or gel [35,36,37]. To confirm that the failure to inhibit biofilm development by *Pseudomonas* was specific to the BPA coating and not due to BZK resistance, we repeated the biofilm inhibition experiments on UI-092-PA and added NS Bactisure to one-half of the MBfIC of BZK to microtiter wells containing BPA-coated catheter pieces. The BPA-coated catheter pieces alone still failed to prevent biofilm formation, but the addition of one-half of the MBfIC of BZK inhibited UI-092-PA biofilm formation on the coated catheter (Figure S3 in Supplementary Materials). In further support of the possibility that the resistance to BPA is connected to the PA-LPS, we found that isogenic LPS mutants of PA14 lacking the O-antigen (a *wbpM* deletion deficient in O-antigen biosynthesis and a *waaL* deletion mutant lacking the ligase that links the O-antigen to the LPS core [85]) were completely susceptible to BPA (Figure 6b). It is possible that the length of the hydrophobic alkyl residues (paraffinic chains with eight to eighteen carbon atoms) were long enough to affect MRSA, *E. coli*, and *E. cloacae*, but too short to affect *P. aeruginosa* [69]. Another possibility is that the density of the BZK molecules on the BPA-coated catheters was sufficient to kill the enteric Gram-negative bacteria (*E. coli* and *E. cloacae*), but not *P. aeruginosa* [69]. The synergy obtained when NS Bactisure was added to the BPA-coated catheter supports this possibility (Figure S3 and Table S4 in Supplementary Materials).

## 4. Materials and Methods

### 4.1. Bacterial Strains

All urinary isolates (UI) were obtained from patients seen at the TTUHSC Urology Clinic. *E. coli* (UI-033-EsC, UI-038-EsC, UI-061-EsC), *E. cloacae* (UI-095-EnC), *P. aeruginosa* (UI-092-PA, UI-040-PA, UI-051-PA), and methicillin-resistant *S. aureus* (MRSA) (UI-009-MRSA) were used for the majority of the experiments. To analyze the effect of *Pseudomonas* LPS on its resistance to the BPA coating, two additional *P. aeruginosa* UIs (UI-040-PA and UI-051-PA), the *P. aeruginosa* laboratory strain UCBPP-PA14 (PA14) originally isolated from a burn wound [52], the PA14 LPS mutants PA14/MrT7::*PA14_23470* (PA14::*wbpM*) and PA14/MrT7::*PA14_66100* (PA14::*66100*) (http://pa14.mgh.harvard.edu/cgi-bin/pa14/home.cgi) (accessed on 24 October 2022) [54], and the *P. putida* strain (CF2954-PP) isolated from sputum of a cystic fibrosis patient (JA Colmer-Hamood, TTUHSC, Lubbock, TX, USA) were used. Gentamicin (50 μg/mL) was added to cultures of PA14::*wbpM* and PA14::*66100*. Antibiotic susceptibility profiles were analyzed for the first six strains listed above, three additional *S. aureus* isolates (UI-073-SA, UI-078-SA, UI-096-SA), six *Enterococcus faecalis* isolates (UI-031-Efl, UI-065-Efl, UI-080-Efl, UI-090-Efl, UI-099-Efl, UI-100-Efl), and two *E. faecium* isolates (UI-020-Efm, UI-048-Efm).

### 4.2. Media, and Growth Conditions 

Frozen stock cultures were inoculated into tryptic soy broth (TSB) and incubated overnight at 37 °C ± 0.5 °C with gentle shaking. Aliquots of the overnight cultures were inoculated into fresh TSB and artificial urine media (AUM) to an OD_600_ of 0.02 for subsequent experiments. The AUM was prepared as described by Brooks and Keevil, 1997 [43]. The diluted cultures (OD_600_ 0.02) were further diluted tenfold in TSB or AUM and 10-μL aliquots were spotted in triplicate on TSB agar plates for quantification of colony forming units (CFU). The CFU/mL were calculated by the following formula:

CFU/mL = average number of colonies in 3 spots × dilution factor × 100

to determine a standard inoculum of ~10^4^ CFU/mL for each strain.

#### 4.2.1. Planktonic Growth in TSB and AUM

An amount of 1-mL aliquots of the diluted suspensions calculated to contain an inoculum of ~10^4^ CFU/mL were pipetted into the wells of a 24-well microtiter plate (Costar untreated, Corning, Durham, NC, USA) and the plate was incubated for 24 h with gentle shaking at 37 °C ± 0.5 °C. After 24 h, the medium in each well was serially diluted tenfold and 10-μL aliquots were spotted in triplicate on Luria-Bertani (LB) agar plates to determine the numbers of CFU/mL.

#### 4.2.2. Biofilm Development on Urinary Catheters

Magic3 intermittent catheters (100% silicone) (Bard, Murray Hill, NJ, USA), Dover red rubber urethral catheters (Cardinal Health, Dublin, OH, USA), and Clean Cath polyvinyl chloride (PVC) urethral catheters (Bard) were used for testing of biofilm development. The sterile silicone, rubber, and PVC catheters were aseptically cut into 1.5-cm pieces for the assays. Biofilm development on the surface of the catheter pieces was conducted using the previously described microtiter plate assay [86]. Experiments were done in triplicate. Catheter pieces were placed individually in wells of a 24-well microtiter plate. The inoculum for each strain was prepared as described above in TSB and AUM to yield 10^5^ CFU/mL, and 1 mL was added to wells containing the catheter pieces. The plates were incubated for 24 h with gentle shaking at 37 °C ± 0.5 °C. After 24 h, the catheter pieces were removed to 1.5 mL microcentrifuge tubes and gently washed twice in 1 mL of phosphate-buffered saline (PBS). Catheters were placed into fresh tubes containing 1 mL of PBS, and the tubes were vortexed for 2 min at 3000 rpm to dislodge the biofilms from the catheter pieces. The suspensions were serially diluted tenfold and 10-μL aliquots were spotted in triplicate on LB agar plates to determine the CFU/catheter piece.

### 4.3. Coating of Silicone Urinary Catheters with the Biofilm Preventative Agent (BPA)

Sterile 1.5-cm lengths of the silicone catheters were washed for two days in sterile PBS. After washing, the catheter pieces were rinsed in sterile water and dried at room temperature. The washed catheter pieces were aseptically transferred to a Petri dish and the dish was sealed and mailed to Next Science (Jacksonville, FL, USA) for coating. The novel biofilm preventative agent BPA, which contains 0.175% BZK (*63349-41-2*), 1.99% polyacrylic acid (PAA) (*9003-01-4*), 2.62% glutaraldehyde (10.47% of 25% glutaraldehyde solution in water) (*110-30-8*) (all from MilliporeSigma, St. Louis, MO, USA), and 95.215% sterile water for irrigation USP (*7732-18-5*) (B. Braun Medical, Bethlehem, PA, USA) (personal communication from Ethan Sherman, Next Science), was applied on both the inner and outer surface of the catheter using the simple dipping method. The PAA was mixed with water in a 100 mL flask using a stir bar for 20 min until the PAA was solubilized. Following PAA solubilization, glutaraldehyde solution was added and mixed for 5 min until combined. Finally, the BZK was added to the solution and mixed until the solution cleared. For catheter coating, individual 1.5-cm catheter pieces were dipped into the solution and allowed to soak for 5 min. The catheters were then removed from the solution and strung up to air dry at room temperature for 24 h. Following drying, the samples were re-dipped into the solution for 5 min and air dried again for 24 h (2× coating). After coating, the pieces were returned to our laboratory for testing. Efficacy of the BPA-coating in preventing biofilm formation on the catheter pieces was evaluated using the same method used to develop biofilms on untreated urinary catheters (above).

### 4.4. Confocal Scanning Laser Microscopy

Biofilms formed on catheter pieces by UI-033-EsC and UI-038-EsC in TSB and AUM were stained using a LIVE/DEAD™ *Bac*Light™ Bacterial Viability kit (Invitrogen, Thermo Fisher Scientific, Waltham, MA, USA). The kit contained two fluorescent dyes, SYTO 9 that stains cells with intact membranes green, and propidium iodide that stains cells with damaged membranes red. Catheter pieces were removed from the microtiter plate and placed in microcentrifuge tubes containing 1 mL of 0.85% NaCl and gently washed twice with an additional 0.85% NaCl. After washing, catheters were placed in 1 mL of 0.85% NaCl, 1.5 μL of SYTO 9, and 2 μL of propidium iodide were added, and the catheters were incubated for 20 min at room temperature in the dark. The catheter pieces were removed from the staining solution and washed twice in 0.85% NaCl, gently blotted on Kimwipes (Kimtech Science, Roswell, GA, USA) to remove excess liquid, and placed in a Petri dish to be visualized. Biofilms on the catheter pieces were visualized using a Nikon T*i*-E microscope with A1 confocal and STORM super-resolution modules. (Nikon Instruments, Melville, NY, USA). Images were obtained through a 10X objective with 525/488 and 595/562 filters for SYTO 9 and propidium iodide, respectively. Image stacks were acquired at 2 μm intervals for 200 steps and three-dimensional image reconstructions were carried out using NIS-Elements 5.21.03 (Nikon Instruments). All instrument settings were consistent for each imaging session.

### 4.5. Statistical Analysis

GraphPad Prism version 9.4.0 (673) (GraphPad Software, San Diego, CA, USA) was used for all statistical analyses. The CFU data were routinely log transformed prior to graphing and statistical analysis. One-way ANOVA was used for comparison of multiple isolates with each other, the three types of catheter material with each other, and the effect of catheter material type on biofilm formation by individual isolates. Tukey’s multiple comparisons posttest was applied to all pairs of pairs or to selected relevant pairs. Unpaired *t* tests were used to compare individual pairs. Data represent the means ± SEM of three independent experiments for each group (*n* = 3). Statistical significance is shown as *, *p* < 0.05; **, *p* < 0.01; ***, *p* < 0.001; ****, *p* < 0.0001; ns, no significant difference.

## 5. Conclusions

In this study, we have described BPA as a novel broad spectrum biofilm preventative catheter coating. BPA-coated catheters prevented biofilm development by *E. coli*, *E. cloacae*, and MRSA, suggesting that this coating provides a broad-spectrum approach to the prevention of CAUTI. Further studies will be required to determine the specific mechanisms by which *P. aeruginosa* resisted killing by the BPA coating, and to discover a way to prevent this loss of efficacy during the coating process. 

## Figures and Tables

**Table 1:** Antibiotic susceptibility patterns of the five Gram-negative uropathogens.

Antibiotic	MIC in μg/mL *				
	*E. coli*			*E. cloacae*	*P. aeruginosa*
	UI-033-EsC	UI-038-EsC	UI-061-EsC	UI-095-EnC	UI-092-PA
Amikacin	≤4, S *	>32, R *	≤4, S	≤4, S	≤4, S
Amoxicillin/clavulanate	16/8, R	8/4, S	8/4, S	>16/8, R	†
Ampicillin	>16, R	>16, R	>16, R	>16, R	†
Aztreonam	>16, R	>16, R	>16, R	>16, R	≤1, S
Cefazolin	>32, R	>32, R	>32, R	>32, R	†
Cefepime	>16, R	16, R	16, R	4, I *	1, S
Cefoxitin	16, I	16, I	≤4, S	>16, R	†
Ceftazidime	>16, R	>16, R	8, R	>16, R	1, S
Ceftriaxone	>32, R	>32, R	>32, R	>32, R	†
Cefuroxime	>16, R	>16, R	>16, R	>16, R	†
Ciprofloxacin	>2, R	>2, R	>2, R	>2, R	2, I
Gentamicin	>8, R	>8, R	≤1, S	1, S	≤0.5, S
Imipenem	≤0.25, S	≤0.25, S	≤0.25, S	‡	8, R
Levofloxacin	>4, R	>4, R	>4, R	>4, R	4, I
Meropenem	≤0.125, S	≤0.125, S	≤0.125, S	≤0.125, S	1, S
Moxifloxacin	>4, R	>4, R	>4, R	>4, R	‡
Nalidixic Acid	>32, R	>32, R	>32, R	§	§
Nitrofurantoin	≤16, S	≤16, S	≤16, S	>64, R	†
Piperacillin/tazobactam	8/4, S	4/4, S	8/4, S	>64/4, R	≤2/4, S
Tetracycline	>8, R	>8, R	>8, R	>8, R	†
Tigecycline	≤0.5, S	≤0.5, S	≤0.5, S	2, S	‡
Tobramycin	>8, R	>8, R	>8, R	1, S	≤0.5, S
Trimethoprim/sulfamethoxazole	≤0.5/9.5, S	>2/38, R	>2/38, R	>2/38, R	†

* MIC: the minimum inhibitory concentration of each antibiotic was determined by the University Medical Center Clinical Microbiology Laboratory; S, susceptible; I, intermediate (light orange); R, resistant (orange); † Antibiotic not tested as *P. aeruginosa* is innately resistant; ‡ Antibiotic not tested by laboratory; § Antibiotic not tested as relevant only for *E. coli.*

**Table 2:** Antibiotic susceptibility pattern of UI-009-MRSA.

Antibiotic	MIC in μg/mL *
Clindamycin	iCR †
Daptomycin	≤1, S
Erythromycin	iCR
Gentamicin	≤1, S
Levofloxacin	≤1, S
Linezolid	2, S
Nitrofurantoin	≤16, S
Oxacillin	>2, R
Penicillin	>1, R
Quinupristin/dalfopristin	≤0.5, S
Rifampin	≤0.5, S
Tetracycline	≤0.5, S
Trimethoprim/sulfamethoxazole	≤0.5/9.5, S
Vancomycin	1, S

* MIC: the minimum inhibitory concentration of each antibiotic was determined by the University Medical Center Clinical Microbiology Laboratory; S, susceptible; R, resistant (orange); † iCR, inducible clindamycin resistance [42].

## Data Availability

The original contributions presented in the study are included in the article/supplementary material, further inquiries can be directed to the corresponding author.

## Supplementary Materials

The following supporting information can be downloaded at: https://www.mdpi.com/article/10.3390/antibiotics11111514/s1, Figure S1. Biofilm biomass formed by the UIs was consistently and most significantly reduced when silicone catheter pieces were incubated in AUM compared to TSB. Figure S2. BPA-coating inhibits biofilm formation by UI-038-ESC on the inner and outer surfaces of the coated catheter. Table S1. Antibiotic resistance patterns of the Gram-negative uropathogens. Table S2. Antibiotic resistance patterns of *S. aureus* urinary isolates. Table S3. Antibiotic resistance patterns of *Enterococcus* spp. urinary isolates. Table S4. MBC and MBfIC of BZK in NS Bactisure. Figure S3. NS Bactisure solution and BPA-coated catheters synergize to prevent UI-092-PA biofilm formation. Methods S1. Minimum Bactericidal Concentration (MBC) of Benzalkonium Chloride (BZK) in NS Bactisure. Methods S2. Minimum Biofilm Inhibition Concentration (MBfIC) of Benzalkonium Chloride in NS Bactisure. References [87,88,89,90] are only cited in Supplementary Materials.
