# Supplementary material for: Urinary Catheters Coated with a Novel Biofilm Preventative Agent Inhibit Biofilm Development by Diverse Bacterial Uropathogens
Source: Antibiotics (Basel). 2022 Oct 30;11(11):1514. doi: 10.3390/antibiotics11111514 (PMC9686518; doi:10.3390/antibiotics11111514)
Supplement: Supplementary file 1 [file antibiotics-11-01514-s001.zip › antibiotics-1975597-supplementary.pdf]

Supplementary Materials

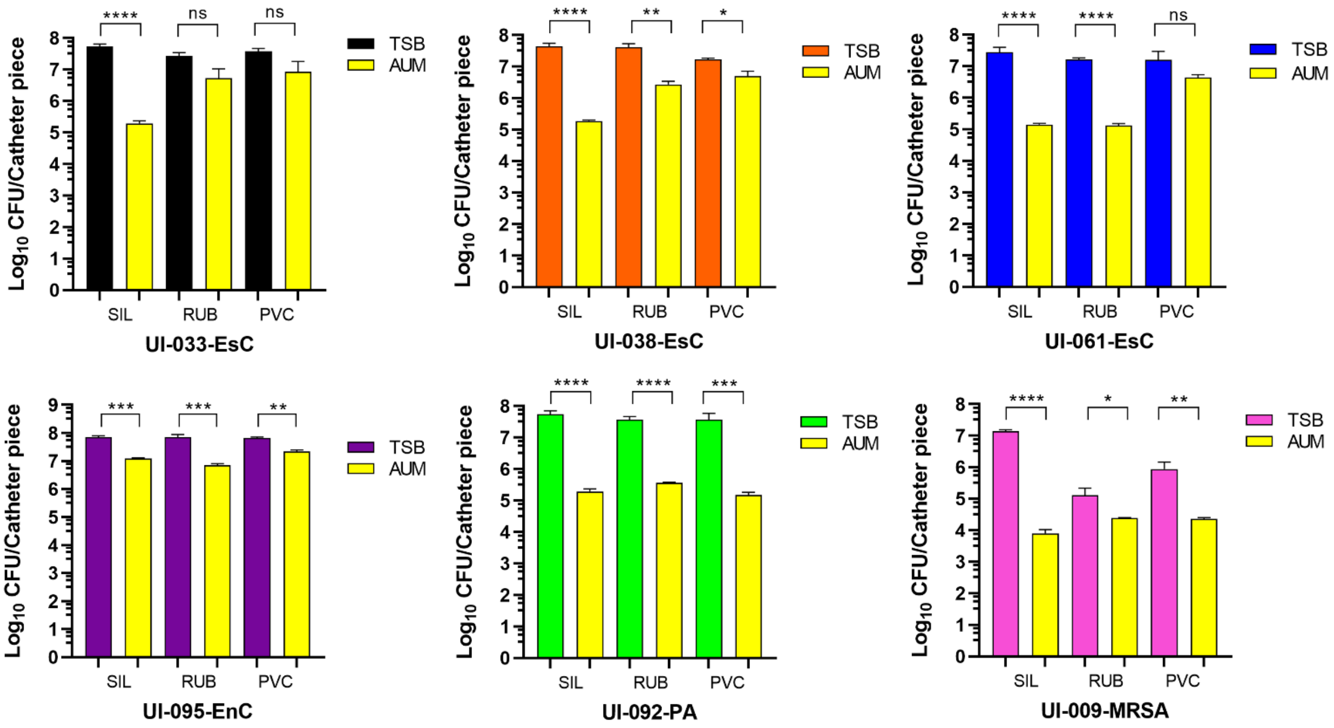

Figure S1. Biofilm biomass formed by the UIs was consistently and most significantly reduced when silicone catheter pieces were incubated in AUM (yellow bars) compared to TSB (black, orange, blue, purple, green, and pink bars). Biofilms were allowed to develop for 24 h on 1.5-cm lengths of urinary catheters made of silicone, rubber, or PVC incubated in TSB or AUM. Bars represent the average of three independent experiments ± SEM; ns, no significance; *, p <0.05; **, p <0.01; ***, p <0.001, ****, p <0.0001.

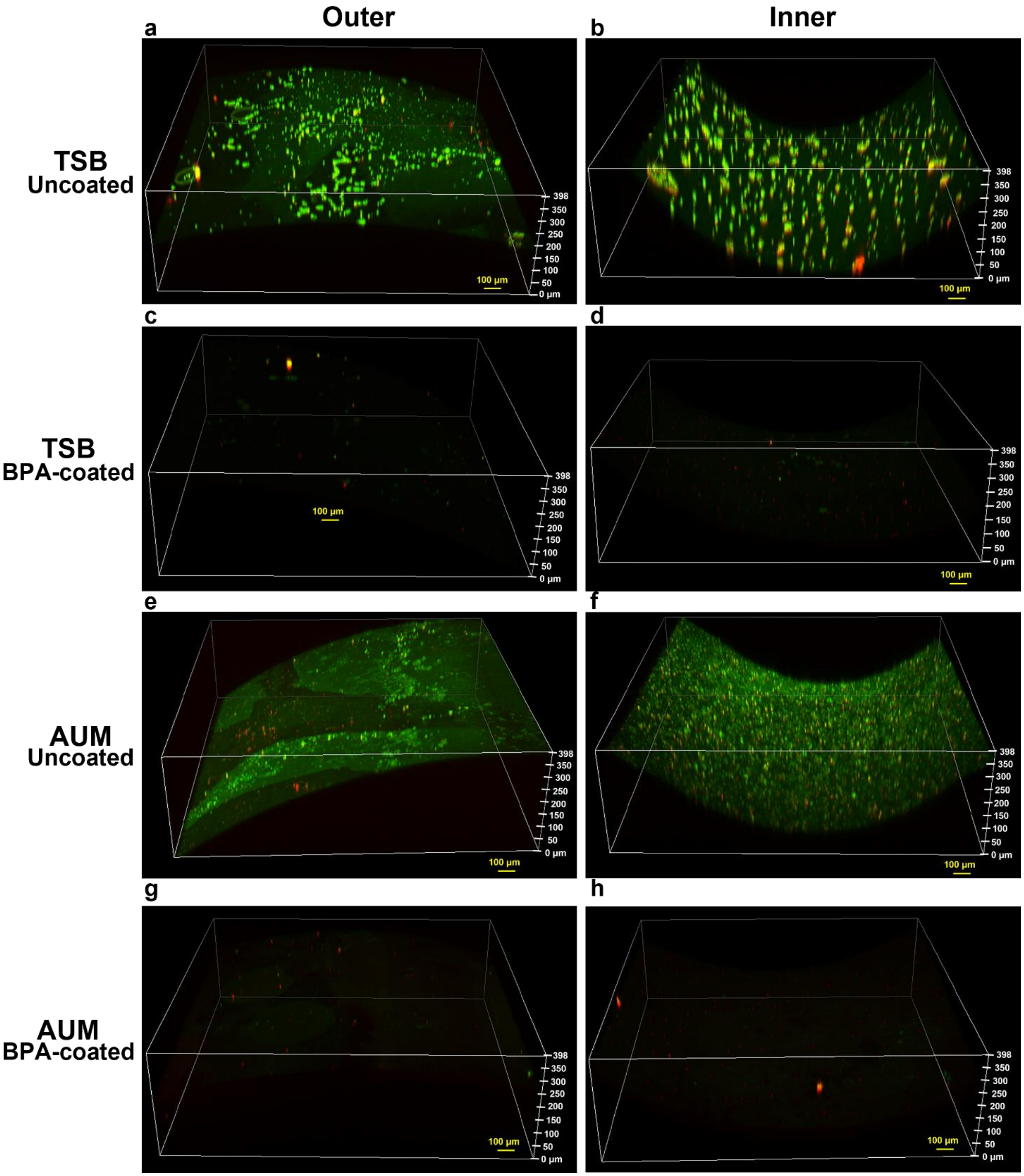

Figure S2. BPA-coating inhibits biofilm formation by UI-038-ESC on the inner and outer surfaces of the coated catheter. Biofilm development was visualized using CSLM following staining with the LIVE/DEAD *Bac*Light™ Bacterial Viability kit; live bacteria are stained green and dead bacteria are stained red. Outer **(a)** and inner **(b)** surfaces of uncoated catheters and outer **(c)** and inner **(d)** surfaces of BPA-coated catheters incubated in TSB. Outer **(e)** and inner **(f)** surfaces of uncoated catheters and outer **(g)** and inner **(h)** surfaces of BPA-coated catheters incubated in AUM. Yellow bars = 100 µm; images are representative of three independent experiments.

Table S1. Antibiotic resistance patterns of the Gram-negative uropathogens .

Antibiotic Class	Antibiotic	MIC in µg/mL*				
		E.coli UI-033-EsC	*E.coli* UI-038-EsC	*E.coli* UI-061-EsC	*E. cloacae* UI-095-EnC	*P. aeruginosa* UI-092-PA
Aminoglycosides	Amikacin	≤4, S*	>32, R*	≤4, S	≤4, S	≤4, S
	Gentamicin	>8, R	>8, R	≤1, S	1, S	≤0.5, S
	Tobramycin	>8, R	>8, R	>8, R	1, S	≤0.5, S
Carbapenems	Imipenem	≤0.25, S	≤0.25, S	≤0.25, S	†	8, R
	Meropenem	≤0.125, S	≤0.125, S	≤0.125, S	≤0.125, S	1, S
Cephalosporins	Cefazolin	>32, R	>32, R	>32, R	>32, R	‡
	Cefepime	>16, R	16, R	16, R	4, I*	1, S
	Cefoxitin	16, I	16, I	≤4, S	>16, R	‡
	Ceftazidime	>16, R	>16, R	8, R	>16, R	1, S
	Ceftriaxone	>32, R	>32, R	>32, R	>32, R	‡
	Cefuroxime	>16, R	>16, R	>16, R	>16, R	‡
Fluoroquinolones	Ciprofloxacin	>2, R	>2, R	>2, R	>2, R	2, I
	Levofloxacin	>4, R	>4, R	>4, R	>4, R	4, I
	Moxifloxacin	>4, R	>4, R	>4, R	>4, R	†
	Nalidixic Acid	>32, R	>32, R	>32, R	§	§
Glycylglycines	Tigecycline	≤0.5, S	≤0.5, S	≤0.5, S	2, S	†
Monobactams	Aztreonam	>16, R	>16, R	>16, R	>16, R	≤1, S
Nitrofurans	Nitrofurantoin	≤16, S	≤16, S	≤16, S	>64, R	‡
Penicillins	Amoxicillin/Clavulanate	16/8, R	8/4, S	8/4, S	>16/8, R	‡
	Ampicillin	>16, R	>16, R	>16, R	>16, R	‡
	Piperacillin/Tazobactam	8/4, S	4/4, S	8/4, S	>64/4, R	≤2/4, S
Sulfonamides	Trimethoprim/ Sulfamethoxazole	≤0.5/9.5, S	>2/38, R	>2/38, R	>2/38, R	‡
Tetracyclines	Tetracycline	>8, R	>8, R	>8, R	>8, R	‡
**Classes tested**	(Antibiotics tested)	10 (23)	10 (23)	10 (23)	10 (21)	6 (11)
**Multidrug Resistance**	MDR	Yes	Yes	Yes	Yes	Yes
	Classes / antibiotics‖	6 / 16	7 / 17	7 / 15	7 / 16	2 / 3
	Percentage resistant	60 / 69.6	70 / 73.9	70 / 65.2	70 / 76.2	33.3 / 27.3

*MIC: the minimum inhibitory concentration of each antibiotic was determined by the University Medical Center Clinical Microbiology Laboratory; S, susceptible; I, intermediate; R, resistant; Antibiotics that are resistant or intermediate are shaded orange

†Antibiotic not tested by laboratory

‡Antibiotic not tested as *P. aeruginosa* is innately resistant

§Antibiotic not tested as relevant only for *E. coli*

‖Number of classes with one or more drugs R or I; number of antibiotics R or I

Table S2. Antibiotic resistance patterns of *S. aureus* urinary isolates.

Antibiotic Class	Antibiotic	MIC in µg/mL*			
		UI-009-MRSA†	UI-073-SA†	UI-078-SA	UI-096-SA
Aminoglycosides	Gentamicin	≤1, S	≤1, S	≤1, S	≤1, S
Cyclic lipopeptides	Daptomycin	≤1, S	≤1, S	≤1, S	≤1, S
Fluoroquinolones	Levofloxacin	≤1, S	>4, R	>4, R	>4, R
		‡	2,S	4,I	4,I
Glycopeptide	Vancomycin	1, S	<0.5, S	<0.5, S	1,S
Macrolides	Clindamycin	iCR§	>2, R	>2, R	<0.5, S
	Erythromycin	iCR	>4, R	>4, R	>4, R
Nitrofurans	Nitrofurantoin	≤16, S	64, I	64, I	≤16, S
Oxazolidinones	Linezolid	2, S	≤1, S	≤1, S	2,S
Penicillins	Oxacillin	>2, R	<=0.25, S	<=0.25, S	0.5,S
	Penicillin	>1, R	>1, R	>1, R	≤0.125, S
Rifamycins	Rifampin	≤0.5, S	<0.5, S	<0.5, S	<0.5, S
Streptogramins	Quinupristin/ Dalfopristin	≤0.5, S	<0.5, S	<0.5, S	<0.5, S
Sulfonamides	Trimethoprim/ Sulfamethoxazole	≤0.5/9.5, S	≤0.5/9.5, S	≤0.5/9.5, S	≤0.5/9.5, S
Tetracyclines	Tetracycline	≤0.5, S	≤0.5, S	≤0.5, S	≤0.5, S
**Classes tested**	(Antibiotics tested)	12 (15)	12 (16)	12 (16)	12 (16)
**Multidrug Resistant**	Classes with R or I / antibiotics R or I	Yes 2 / 4	Yes 4 / 5	Yes 4 / 5	No 2 / 2

*MIC: the minimum inhibitory concentration of each antibiotic was determined by the University Medical Center Clinical Microbiology Laboratory; S, susceptible; I, intermediate; R, resistant; Antibiotics that are resistant or intermediate are shaded orange

†MRSA, methicillin *Staphylococcus aureus*; SA, *S. aureus*

‡Antibiotic not tested by laboratory

§iCR, inducible clindamycin resistance [1]

Table S3. Antibiotic resistance patterns of *Enterococcus* spp. urinary isolates.

Antibiotic Class	Antibiotic	MIC in µg/mL*							
		UI-031-Efl†	UI-065-Efl	UI-080-Efl	UI-090-Efl	UI-099-Efl	UI-100-Efl	UI-020-Efm†	UI-048-Efm
Aminoglycosides	Gentamicin	≤500,S	≤500,S	≤500,S	≤500,S	≤500,S	≤500,S	≤500,S	>500, R
	Streptomycin	≤1000,S	>1000, R	≤1000,S	≤1000,S	≤1000,S	≤1000,S	≤1000,S	≤1000,S
Cyclic lipopeptides	Daptomycin	2,S	≤1, S	2,S	2,S	2,S	4,S	≤1, S	2,S
Fluoroquinolones	Levofloxacin	≤1,S	>4, R	≤1,S	≤1,S	≤1,S	≤1,S	≤1,S	>4, R
Glycopeptide	Vancomycin	1,S	1,S	2,S	1,S	1,S	1,S	≤0.5, S	≤0.5, S
Nitrofurans	Nitrofurantoin	≤16, S	≤16, S	≤16, S	≤16, S	≤16, S	≤16, S	‡	‡
Oxazolidinones	Linezolid	≤1,S	≤1,S	≤1,S	≤1,S	≤1,S	2,S	2,S	2,S
Penicillins	Ampicillin	1, S	2,S	2,S	1, S	0.5, S	1, S	1, S	>8,R
	Penicillin	2,S	4,S	8,S	4,S	≤1,S	4,S	2,S	>8,R
Tetracyclines	Tetracycline	≤0.5, S	>8, R	≤0.5, S	>8, R	>8, R	>8, R	≤0.5, S	>8, R
**Classes tested**	(Antibiotics tested)	8 (10)	8 (10)	8 (10)	8 (10)	8 (10)	8 (10)	7 (9)	7 (9)
**Multidrug Resistance**	MDR	No	Yes	No	No	No	No	No	Yes
	Classes /antibiotics§	0 / 0	3 / 3	0 / 0	1 / 1	1 / 1	1 / 1	0 / 0	4 / 5
	Percentage resistant	0 / 0	37.5 / 30	0 / 0	12.5 / 10	12.5 / 10	12.5 / 10	0 / 0	57 / 55.6

*MIC: the minimum inhibitory concentration of each antibiotic was determined by the University Medical Center Clinical Microbiology Laboratory; S, susceptible; I, intermediate; R, resistant; Antibiotics that are resistant or intermediate are shaded orange

†Efc, *Enterococcus faecalis*; Efm, *E. faecium*

‡Antibiotic not tested by laboratory

§Number of classes with one or more drugs R or I; number of antibiotics R or I

Methods S1. Minimum Bactericidal Concentration (MBC) of Benzalkonium Chloride (BZK) in NS Bactisure

The MBC of the BZK in NS Bactisure wound lavage (Zimmer Biomet, Warsaw, IN, USA) was determined using the broth dilution method as previously described [2-4]. Urinary isolates (UIs) were grown overnight in tryptic soy broth (TSB) at 37°C with gentle shaking. Aliquots of the overnight cultures grown in TSB were inoculated into fresh TSB or artificial urine medium (AUM) to an OD_{600} of 0.02 for subsequent experiments. The AUM was prepared as described by Brooks and Keevil, 1997 [5]. The diluted cultures (OD_{600} 0.02) were further diluted tenfold in TSB or AUM and 10-μL aliquots were spotted in triplicate on TSB agar plates for quantification of colony forming units (CFU). To determine a standard inoculum of ~10^5 CFU/mL for each strain, the CFU/mL were calculated by the following formula:

$$\text{CFU/mL} = \text{average number of colonies in 3 spots} \times \text{dilution factor} \times 100$$

For the MBC, NS Bactisure was serially diluted twofold in TSB or AUM for a range of 0.63 to 162.5 μg/mL of BZK as the active ingredient and a 1-mL aliquot of each dilution was pipetted in triplicate into wells of a 24-well microtiter plate and inoculated with 10 μL of overnight culture diluted to OD_{600} 0.02 to obtain an initial inoculum of 10^5 CFU/mL. As a growth control, three wells with medium alone were inoculated with each strain; as a sterility control, one well contained medium alone. The microtiter plate was incubated for 24 h at 37°C with gentle shaking. The minimum inhibitory concentration of BZK for each UI was recorded as the lowest concentration where no growth was visually observed. The growth control well was serially diluted tenfold, and aliquots of each dilution were spotted in triplicate on TSB to determine the growth without antimicrobial solution. One hundred μL aliquots from the well with the MIC for each isolate and all wells with higher concentrations of BZK were plated in triplicate on TSB agar plates; all the plates were incubated overnight. The MBC was considered the lowest concentration that demonstrated a 99.99% reduction in CFU/mL when compared to the MIC dilution:

$$\frac{\left(\frac{\text{CFU}}{\text{well}} \text{ x } 100\right)}{\text{CFU control}} = \text{surving bacteria x } 10 = \% \text{ surviving bacteria}$$

$$100\% - \% \text{ surviving bacteria} = \% \text{ reduction in bacterial load}$$

Methods S2. Minimum Biofilm Inhibition Concentration (MBfIC) of BZK in NS Bactisure

The Calgary biofilm device (CBD) assay was utilized as previously described with modifications [6] to determine the MBfIC of BZK in NS Bactisure. Microtiter plates were prepared as described above for determination of MBC using AUM for the medium, and uncoated silicone catheter pieces (1 inch) were placed in all wells. The wells were inoculated with UIs as for the MBC (except for the sterility control) and the plates were incubated overnight at 37°C with gentle shaking. After 24 h, the catheter pieces were removed to 1.5 mL microcentrifuge tubes and washed gently twice in 1 mL of phosphate buffered saline (PBS). Catheters were placed into fresh 1.5 mL tubes containing 1 mL of PBS, and the tubes were vortexed for 2 min at 3000 rpm to dislodge the biofilms from the catheter pieces. The suspensions were serially diluted tenfold and 100-μL aliquots were plated in triplicate on TSB agar plates to determine the CFU/catheter piece. The lowest concentration of BZK that inhibited 99.99% of growth on the catheter pieces was considered the MBfIC.

Table S4. MBC and MBfIC of BZK in NS Bactisure.

Isolate	Concentration of BZK (μg/mL)		
	TSB	AUM	AUM
	MBC	MBC	MBfIC*
UI-033-EsC	20.31†	40.63	40.63
UI-038-EsC	20.31	40.63	40.63
UI-061-EsC	20.31	20.31	20.31
UI-095-EnC	20.31	40.63	40.63
UI-092-PA	81.25	81.25	81.25
UI-009-MRSA	1.27	2.54	2.54

AUM, artificial urine medium; BZK, benzalkonium chloride; MBC, minimum bactericidal concentration; MBfIC, minimum biofilm inhibition concentration; TSB, tryptic soy broth

*Prevention of biofilm formation on uncoated silicone catheters (see Methods S1 and S2)

†Values are averages of three individual experiments

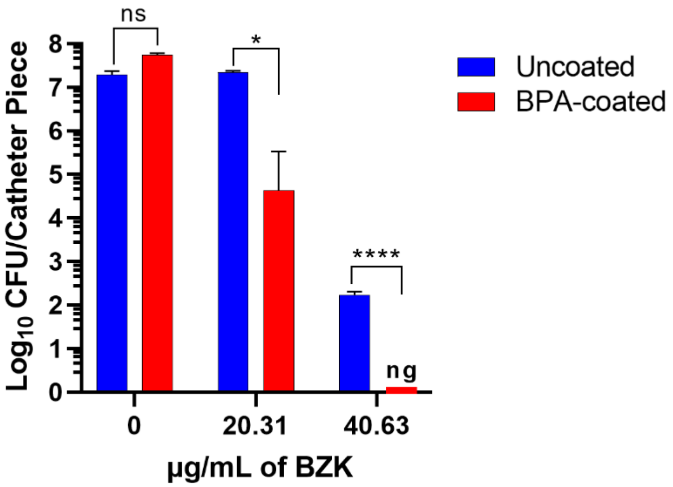

Figure S3. Benzalkonium chloride (BZK) in NS Bactisure and BPA-coated catheters synergize to prevent UI-092-PA biofilm formation. Uncoated or BPA-coated catheters were placed in 1 mL AUM containing twofold dilutions of NS Bactisure calculated in μg/mL of the active ingredient BZK and inoculated with 10^5 CFU of UI-092-PA. Plates were incubated for 24 h and the catheter pieces were removed to 1.5 mL microcentrifuge tubes and washed gently twice in 1 mL of PBS. Catheters were placed into fresh 1.5 mL tubes containing 1 mL of PBS, and the tubes were vortexed for 2 min at 3000 rpm to dislodge the biofilms from the catheter pieces. The suspensions were serially diluted tenfold and 10-μL aliquots were spotted in triplicate on TSB agar plates to determine the CFU/catheter piece. The CFU data was log transformed prior to graphing and statistical analysis. Unpaired *t* tests were used to compare individual pairs (GraphPad Prism version 9.4.0 (673); GraphPad Software, San Diego, CA, USA). Bars represent the means ± SEM of three independent experiments for each group (n = 3). Statistical significance is shown as *, $p < 0.05$; ****, $p < 0.0001$; ns, no significant difference. The addition of one-half the MBfIC of BZK in NS Bactisure to the BPA-coated catheter prevented biofilm formation by UI-092-PA (Table S4).

References S1

1. Timsina, R.; Shrestha, U.; Singh, A.; Timalsina, B. Inducible clindamycin resistance and *erm* genes in *Staphylococcus aureus* in school children in Kathmandu, Nepal. *Future Sci OA* **2020**, *7*, FSO361, doi:10.2144/fsoa-2020-0092.
2. Andrews, J.M. Determination of minimum inhibitory concentrations. *J Antimicrob Chemother* **2001**, *48 Suppl 1*, 5-16, doi:10.1093/jac/48.suppl_1.5.
3. Balouiri, M.; Sadiki, M.; Ibnsouda, S.K. Methods for in vitro evaluating antimicrobial activity: A review. *J Pharm Anal* **2016**, *6*, 71-79, doi:10.1016/j.jpha.2015.11.005.
4. Wiegand, I.; Hilpert, K.; Hancock, R.E. Agar and broth dilution methods to determine the minimal inhibitory concentration (MIC) of antimicrobial substances. *Nat Protoc* **2008**, *3*, 163-175, doi:10.1038/nprot.2007.521.
5. Brooks, T.; Keevil, C.W. A simple artificial urine for the growth of urinary pathogens. *Lett Appl Microbiol* **1997**, *24*, 203-206.
6. Ceri, H.; Olson, M.E.; Stremick, C.; Read, R.R.; Morck, D.; Buret, A. The Calgary Biofilm Device: new technology for rapid determination of antibiotic susceptibilities of bacterial biofilms. *J Clin Microbiol* **1999**, *37*, 1771-1776, doi:10.1128/JCM.37.6.1771-1776.1999.
